# Mental Illness-Related Stigma Among University Students, Medical Professionals, and Allied Health Workers in the United Arab Emirates

**DOI:** 10.1192/bjo.2026.11067

**Published:** 2026-06-30

**Authors:** Mohammed Al Ahbabi, Fadwa Al Mugaddam, Gabriel Andrade, Syed Fahad Javaid

**Affiliations:** 1 Behavioural Sciences Institute, Al Ain, UAE; 2 Department of Psychiatry, College of Medicine and Health Sciences, United Arab Emirates University, Al Ain, UAE; 3 Ajman University, Ajman, UAE

## Abstract

**Aims:**

Stigma involves the devaluation of individuals based on personal attributes and can lead to discrimination, social exclusion, and poorer mental health outcomes, strongly shaped by cultural and social norms. The United Arab Emirates (UAE) provides a unique context to examine mental illness stigma, particularly among university students and healthcare-related groups, where stigma may hinder help-seeking and care delivery. This study aimed to quantify levels of mental illness-related stigma and examine socio-demographic and occupational correlates among university students, medical professionals and allied health workers in the UAE.

**Methods:**

We conducted a cross-sectional, questionnaire-based study using the nine-item Stigma-9 Questionnaire (STIG-9) to assess perceived public stigma towards people with mental illness (total score 0–27; higher scores indicate greater stigma). Participants were recruited via non-probability sampling at a federal university and multiple tertiary hospitals in the UAE, using email invitations to participate in an anonymous online survey. Demographic variables included gender, age group, nationality, continent of origin, marital status, religion, family income, caregiver experience, occupation/status, type of activity and place of study/work. Descriptive statistics summarised sample characteristics and STIG-9 scores. Independent-samples t-tests and one-way ANOVA with Tukey post hoc tests compared mean stigma scores across groups; significance was set at p<0.05.

**Results:**

A total of 510 participants completed the questionnaire; 70.4% were female, and 48.0% were Emirati. Most were aged 16–25 (43.5%) or 31–40 (24.9%). 42.2% were healthcare providers or administrators, 40.0% were undergraduate students, and 17.8% were postgraduate students. The mean STIG-9 score was 17.5 (SD 5.64), indicating substantial perceived stigma in this educated cohort. Age was significantly related to stigma (F(4,505)=2.52, p=0.04): 16–25-year-olds reported higher stigma than 31–40-year-olds (mean difference 1.81, p=0.031). Emirati participants scored higher than non-Emiratis (t(508)=2.33, p=0.02, Cohen’s d=0.21). No significant differences in stigma were found based on gender, nationality, marital status, religion, income, caregiver experience, occupation, study/work location, or when comparing medical vs. non-medical students, undergraduate vs. postgraduate students, or healthcare groups.

**Conclusion:**

Mental illness stigma is common among university students and healthcareprofessionals in the UAE, despite high education levels. Higher stigma among younger adults and Emirati nationals indicates cultural and generational influences. Culturally informed anti-stigma education in universities and healthcare training, especially for younger Emiratis, could reduce stigma, enhance help-seeking, and promote equitable mental healthcare.

No financial sponsorship has been received for this study.

